# Minimal training sufficient to diagnose pediatric wrist fractures with ultrasound

**DOI:** 10.1186/s13089-017-0066-z

**Published:** 2017-05-08

**Authors:** Henrik Hedelin, Christian Tingström, Hanna Hebelka, Jon Karlsson

**Affiliations:** 1 Department of Orthopedics, Sahlgrenska University Hospital, Instititute of Clinical Sciences, Sahlgrenska Academy, University of Gothenburg, Gothenburg, Sweden; 2 Department of Radiology, Institute of Clinical Sciences, Sahlgrenska Academy, University of Gothenburg, Gothenburg, Sweden; 3 Barnortopeden Östra sjukhuset, Sahlgrenska Universitetssjukhuset, Smörslottsgatan 1, 416 78 Gothenburg, Sweden

**Keywords:** Ultrasound, Pediatric, Fracture, Emergency, Wrist

## Abstract

**Background:**

In children, non-fractured wrists generally need no treatment and those that are fractured may only require a 3-week cast without any clinical follow-up. The ability to perform a point-of-care triage decision if radiographs are needed could improve patient flow and decrease unnecessary radiographs. The aim of this study was to evaluate the role of ultrasound (US) as a point-of-care triage tool for pediatric wrist injuries with limited training.

**Methods:**

Physicians with no previous US experience attended a 1.5 h course in the use of US to diagnose distal radius fractures at the Emergency Department (ED). The physicians firstly used US to diagnose a potential fracture and, if the patient had a fracture, grouped the patient according to how they wanted him/her to be treated based on US. The physician then interpreted the subsequent radiographs and decided on a treatment based on this information. Consultant traumatologists and a senior radiologist established a gold standard for correct treatment and radiological diagnosis, respectively.

**Results:**

One hundred and sixteen injuries in 115 patients were included. The ED physician identified 75 fractures on radiographs. With the exception of a minimal buckle fracture, all were identified on US. US had a tendency to interpret complete fractures on radiographs as incomplete (*n* = 7) leading to incorrect treatment decisions.

**Conclusions:**

In the hands of an US novice, US examination is comparable with radiographs as a point-of-care tool to distinguish a fractured wrist from a non-fractured one. US is not, however, as good as radiographs for placing fractured wrists into the correct treatment group.

**Level of Evidence:**

Level III. Diagnostic study of non-consecutive patients.

**Electronic supplementary material:**

The online version of this article (doi:10.1186/s13089-017-0066-z) contains supplementary material, which is available to authorized users.

## Background

At Emergency Departments (ED) children with wrist trauma constitute a large proportion of the admitted patients in all age groups. Approximately 30% of all fractures in children are located in the forearm [1, 2] and the majority of those fractures are in the distal radius. Radiographs are the standard diagnostic tool for pediatric patients with trauma to the wrist. Treatment and acceptable angulations vary greatly depending on the age of the child and the fracture type. Exact guidelines for treatment also vary between individual physicians, hospitals, and countries.

The general use of ultrasound (US) by non-radiologists has increased over the last decade and in many cases focus has shifted toward using US in the ED and in the pre-hospital setting to exclude a specific injury rather than to visualize an entire organ system [3, 4]. In the orthopedic field, the diagnosis of many fracture types have been evaluated [5–10] in this setting and results have been shown to be promising. US as a triage tool for ankle fractures in adults has been shown to greatly decrease the need for radiographs with only minimal training of the involved physicians [11]. A recent systematic review and meta-analysis of the use of US in the diagnosis of distal forearm fractures in children including 1204 patients showed a high accuracy of US as a diagnostic tool with a sensitivity and specificity of 97 and 95%, respectively [12]. The authors conclude that US can safely be used to establish the diagnosis of distal forearm fractures in children. In a sub-group of the meta-analysis, there was no difference in sensitivity based on the experience of the examining physician. However, there is no standardized method to quantify experience. The use of US as a primary screening tool is still under debate.

Ultrasound as a point-of-care triage tool to select the children with wrist trauma who need radiographs could decrease the need for unnecessary radiographs. This would reduce radiation and possibly save resources for the hospital and the average time a patient needs to spend at the ED.

The primary aim of the study was to add information to the current debate by investigating if US can be used as a point-of-care triage tool in the hands of non-radiologists with standardized minimal training to differentiate between a fracture and no fracture. The secondary aim was to evaluate if US can differentiate between complete fractures (usually needing follow-up) and buckle fractures (that can often be treated without a follow-up at the hospital).

## Methods

The Queen Silvia Children’s Hospital (DSBUS) is the only regional hospital for pediatric trauma in the western region of Sweden. On a normal day the orthopedist on call takes care of between 10 and 40 patients. All follow-ups were made at the same hospital’s orthopedic out-patient department.

Six physicians ranging from junior doctors to consultants participated in the inclusion of patients, all without previous experience of US diagnostics.

**Fig. 1:**
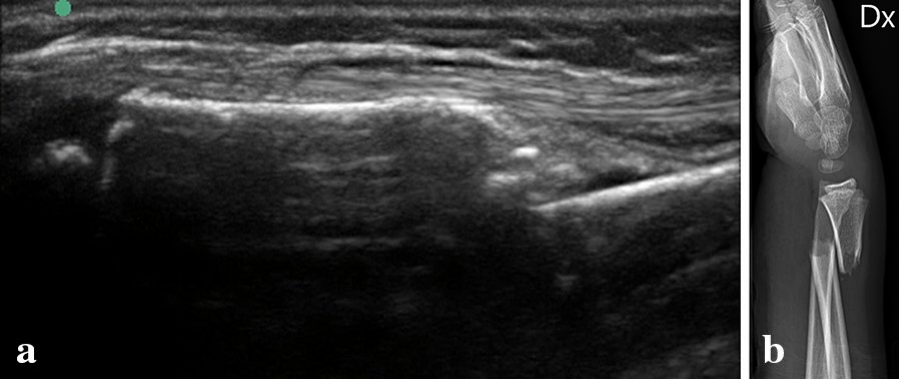
**a** Displaced complete fracture of the distal radius. US dorsal longitudinal view. **b** (Same case as image **a**) Radiograph lateral view. Displaced complete fracture of the distal radius

**Fig. 2:**
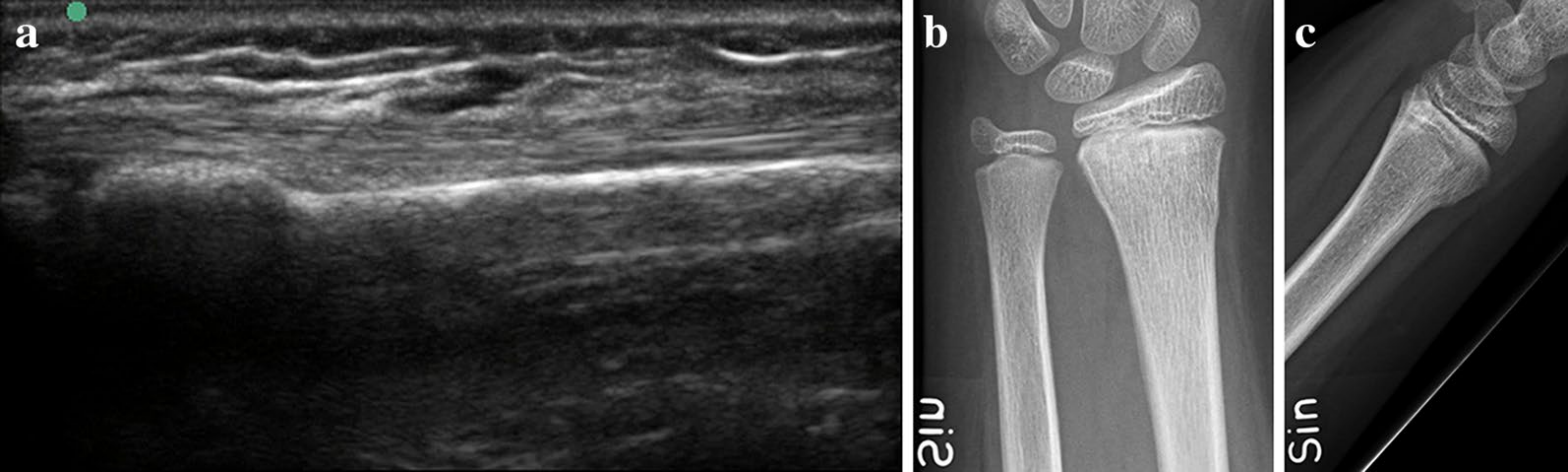
**a** Infraction/torus fracture of the distal radius, US dorsal longitudinal view. **b**, **c** (Same case as **a**) Infraction/torus fracture of the distal radius on lateral and AP radiographs

They attended a 1.5 h intensive course designed by a pediatric radiologist and an orthopedic surgeon (both authors). The course focused only on the diagnosis of suspected wrist fractures in children. The study physicians performed one examination each on a patient with a fracture to verify that they were acquainted with the method. A fracture was diagnosed only if a cortical gap, torus formation, or displacement was seen (Figs. 1, 2). Indirect signs like subperiosteal hematoma was not used since these signs are deemed more subtle and difficult to learn and interpret for an US novice.

When present at the ED during the study period between February and September 2015, study physicians included all patients eligible. The US equipment used was only available for this time period and was only used for the purpose of this study. Inclusion criteria were all patients between 3 and 16 years with wrist pain after recent (last 3 days) trauma. Patients were excluded if there was an uncertain clinical finding (i.e., pain from proximal forearm or hand), an open fracture, or if the parents did not have the language skills to understand the information material.

Distal radial fractures in children can be categorized in a variety of ways such as buckle (or torus) fractures, greenstick fractures, complete fractures, and fractures involving the physis (classified according to the Salter–Harris classification [13]). For the purpose of this study the exact radiological classification was of less importance since the focus was on the effects of the treatment.

At our department the vast majorities of cases are treated with only a splint or closed reduction followed by a splint [2]. The treatment guidelines for the ED doctors at the hospital can, in a simplified manner, be divided into four groups:

1. No fracture.
  Advised treatment: No splint or immobilization.
2. A buckle or greenstick fracture with acceptable angulation.
  Advised treatment: A dorsal forearm plaster of Paris splint for 3 weeks that is removed by the parents. No follow-up is needed.
3. A buckle or greenstick fracture with borderline angulation or complete fracture with minor angulation or displacement.
  Advised treatment: A dorsal forearm splint for 3–4 weeks with a radiographic control of the fracture position after 5–7 days.
4. A buckle or greenstick fracture with unacceptable angulation or complete fractures with little angulation or displacement.
  Advised treatment: Closed reduction with or without surgical intervention.

The term “acceptable angulation” is relative since, as noted above, indications for intervention or follow-up depends on the age of the child and other factors. It is therefore up to the ED physician to make a qualified decision with all factors taken into account.

**Fig. 3:**
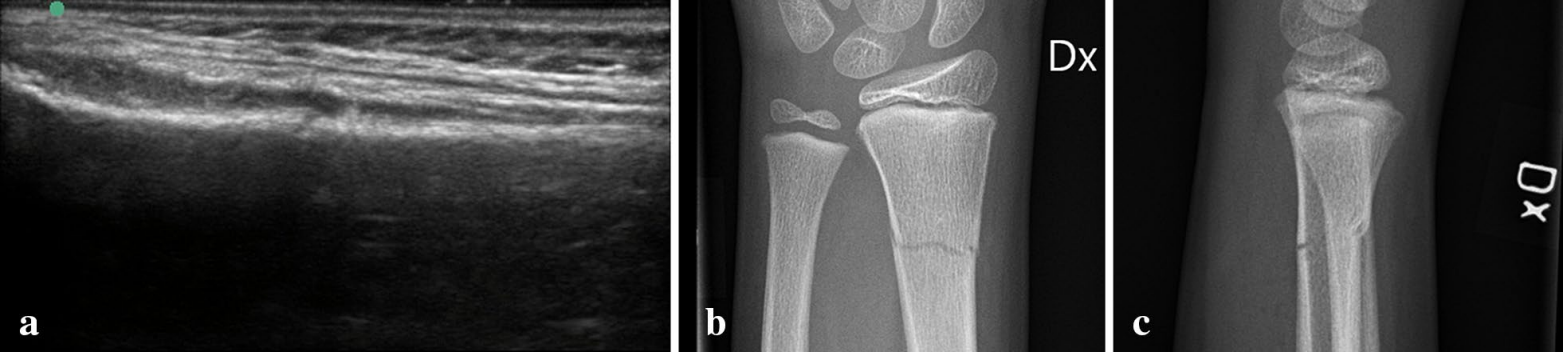
**a** Complete non-displaced fracture of the distal radius. US volar longitudinal view. Note the discontinuous *cortical line*. **b**, **c** (Same case as **a**) Complete non-displaced fracture of the distal radius on lateral and AP radiographs

A radiographic follow-up is thus, according to the guidelines for ED doctors, needed if (a) the angulation or displacement is such that any further angulation is unacceptable or (b) the fracture is potentially unstable. The latter is the case for many complete fractures with even minor displacement (Fig. 3). Rare fractures involving the actual joint (Salter–Harris type 3 or 4) generally require a follow-up radiograph or surgery. No such fractures were found in the present study.

Each patient had a physical examination of the wrist and US of the wrist was performed with a Fujifilm SonoSite, Inc. Edge^®^ using a linear 15–6 MHz probe. The US examination took 5–10 min to perform and consisted of longitudinal images in the sagittal plane of the distal 10 cm of the radius with special focus on painful areas. Dorsal, radial, and volar aspects of the distal radius were visualized. Imaging of the radial portion of the wrist in the sagittal plane on US visualized the same plane as a lateral view on radiographs. The decision of the study physician was stored in a coded closed envelope in order to blind the result from the results of the radiograph. The patient was asked if he or she found the examination painful.

The possible outcomes of the US examination correspond to the treatment groups 1–4 described above. The study physician first grouped each patient based only on the US interpretation. The alternative “uncertain finding” was used when the study physician was not able make a decision based on the US findings alone or could not achieve adequate images.

After the US examination standard radiographs of the wrist, using AP and lateral views, were performed in all patients. The same four outcomes were used. The study physician interpreted the images and stored the results in a second envelope.

To validate the accuracy of the radiographic readings by the study physicians the radiographs were also, at a later time, inspected by an independent senior radiologists blinded to the US and radiograph interpretation of the study physician. The radiologist did not sort the fractures according to the four groups used by the study physicians, since the radiologist were not involved in the decision making of the treatment, and the interpretation was used only to analyze the specific fractures where the two diagnostic modalities did not agree. The senior radiologist did no interpret the US images.

To obtain a gold standard for treatment, two senior consultants in pediatric orthopedic surgery, specialized in fractures, analyzed all the radiographs and decided on the most appropriate treatment. This second look was blinded to all other results and interpretations. The consultants were asked “what is the optimal treatment for a patient of this age with this injury based on the radiograph” with the treatment groups corresponding to numbers 1–4 presented above. The consultants established a joint consensus and there were no cases of dispute. This method was used in order to evaluate the clinical relevance of the results and not limit the study to if the US and radiological findings correlated perfectly.

### Ethics, consent, and permissions

Participation was voluntary but all patients who met the inclusion criteria approved of participation and subsequent publishing of obtained material. Ethical approval was obtained from the Human Ethics Committee at the Medical Faculty, Gothenburg University (DNR 956-14).

### Statistics

To reach the desired level of evidence based on pre-study power analysis, a study group of 130 patients was desirable. The sample size was depending on 80% power and *p* < 0.05 together with normal distribution, estimating that using US 75% of the fractures would be detected. The very conservative 75% sensitivity estimate was chosen based on the lowest sensitivity that was found in relevant studies [14]. However, only 117 cases were included due to the limited time the ultrasound equipment was available. As mentioned in the result section the sensitivity was shown to be clearly higher than the conservative estimate used to estimate power.

## Results

Examinations of 117 injuries in 116 patients were included between February and August 2015. One patient was excluded due to loss of data and therefore 116 injuries in 115 patients (62 female/53 male) were analyzed. The median age was 11 years (range 3–16). The patients were fairly evenly distributed among the study physicians (range 10–43, median 16). None of the patients described the procedure as more than mildly painful.

### Ultrasound by study physician vs radiograph interpretation by study physician (shown in Table 1)

The results of US and radiographic examination are reported in Table 1. Out of 75 fractures seen on radiographs by the study physician, one was interpreted as a non-fractured wrist on US. This misdiagnosed case was interpreted as a minimal dorsal buckle fracture by the radiologists on the radiographs.

**Table 1:** The examination outcomes of the US and radiographs as interpreted by the study physician

		Radiographic assessment by study physician				Total
		No fracture	Buckle fracture/greenstick fracture	Complete fracture or unacceptable angulation	Uncertain finding	
US assessment by study physician	No fracture	27	1	0	2	30
	Buckle fracture/greenstick fracture	4	51	7	2	64
	Complete fracture or unacceptable angulation	0	1	14	0	15
	Uncertain finding	4	1	0	2	7
Total		35	54	21	6	116

The radiologist’s interpretation is not shown here

Seven out of 21 complete fractures were interpreted as a “greenstick or a buckle fracture” on the US examination. All of the fractures in question were, incorrectly, assumed to be greenstick fractures by the study physician. Misinterpreted fractures were evenly distributed among the study physicians.

### Radiographic interpretation by study physician vs radiograph interpretation by radiologist

The senior radiologist identified a total of 79 fractures which illustrates that the radiographic interpretation by the study physician was not completely accurate. Three cases that the ED physicians considered “uncertain findings” on radiographs proved to be fractures. The one fracture that was overlooked on the radiograph by the ED physician was an undisplaced distal tip ulna fracture that was stable but was treated with splint for pain relief. This fracture was also missed on US. The study physician identified all complete or displaced fractures on radiographs but three did not need the follow-up suggested by the study physician. Out of 54 fractures judged by the study physician to be buckle fractures or greenstick fractures, not needing follow-up, five were considered complete fractures by the radiologist. Only three of these needed a follow-up according to the senior traumatologists.

### Radiographic interpretation by study physician vs correct treatment based on radiographs according to senior traumatologists (shown in Table 2)

Two patients that needed a follow-up were missed using radiographs as the tool to inform treatment decision by the study physician. One patient who was judged not to need any treatment needed a cast for 3 weeks according to senior traumatologists.

**Table 2:** The study physician’s interpretation of the radiographs at the ED cross-tabulated with the gold standard treatment decided by senior traumatologists based on radiographs

		Appropriate treatment based on radiographs				Total
		No treatment	Cast for 3 weeks without follow-up	Cast with radiographic follow-up	Reposition or operation needed	
Radiograph assessment by study physician	No fracture	34	1	0	0	35
	Buckle fracture/greenstick fracture	0	52	2	0	54
	Complete fracture or unacceptable angulation	0	3	14	4	21
	Uncertain finding	4	2	0	0	6
Total		38	58	16	4	116

The radiologist’s interpretation is not shown here

### Ultrasound by study physician vs correct treatment based on radiographs according to senior traumatologists (shown in Table 3)

Two patients judged to have no fracture needed a cast for 3 weeks. Five patients believed to have a buckle or greenstick fracture (with acceptable angulation) turned out to need a follow-up (they were complete). One patient with a greenstick fracture needed repositioning according to senior traumatologists despite being grouped as an acceptable angulation by the study physician. Thus a total of eight fractures would not have received necessary treatment using only US as a triage tool to decide treatment. There was also, a tendency to over-treat benign fractures or non-fractured wrists.

**Table 3:** The study physician’s interpretation of the US examination at the ED cross-tabulated with the gold standard treatment decided by senior traumatologists based on radiographs

		Appropriate treatment based on radiographs				Total
		No treatment	Cast for 3 weeks without follow-up	Cast with radiographic follow-up	Reposition or operation needed	
US assessment by study physician	No fracture	28	2	0	0	30
	Buckle fracture/greenstick fracture	5	53	5	1	64
	Complete fracture or unacceptable angulation	0	2	10	3	15
	Uncertain finding	5	2	0	0	7
Total		38	59	15	4	116

### Sensitivity and specificity

Compared with the radiologist’s gold standard the study physicians ability to distinguish a fracture from a non-fracture using US has a sensitivity of 97.4% (95% CI 90.9–99.7%) and a specificity of 84% (95% CI 67.2–94.7%), assuming normal distribution and omitting uncertain findings (Table 4). Since US was evaluated as a triage tool to see if the patient needed a radiograph “uncertain findings” could be included as true positive for the calculation since these cases would have been triaged to need a radiograph. Omitting these cases for the above calculations produces a more conservative estimate of the sensitivity. The isolated stable ulna fissure was not included as a fracture since the distal ulna was not examined using US. Apart from the calculations of sensitivity and specificity above regarding the ability of US to identify the non-fractured wrist, the results regarding the correct treatment of identified fractures are presented only as descriptive statistics.

**Table 4:** Sensitivity and specificity for US to detect any fracture type

	US examination positive	US examination negative	Total
Fracture present	75	2	77 (2 uncertain)
Fracture absent	5	27	32 (5 uncertain)
Total	80	29	116 (77 + 32 + 2 + 5)
	Sensitivity: 97.4% (95% CI 90.9–99.7%)	Specificity: 84% (95% CI 67.2–94.7%)	

## Discussion

The main finding of the current study is that physicians with limited but standardized training can use point-of-care US examination to distinguish distal radius fracture from non-fracture trauma in an age mixed pediatric patient group.

A number of studies on the use of US for forearm fractures in children report approximately 95% sensitivity and specificity for fracture detection, which are in accordance with the results of the present study [10, 12, 14–18]. Some previous studies have included all forearm fractures, leading to lower sensitivity and specificity [14, 19, 20].

With few exceptions previous studies have included experienced ultrasonographers (or do not present the experience of the examiner). US is considered user-dependent and therefore this constitutes a weakness in most studies. What an expert can find is very different from what can be expected to be found by a novice and the ED is generally staffed by physicians with limited US experience. We regard limited training as training that can take place in a few hours requiring only a few sample cases. The studies limited to physicians without any prior experience in US and examining only wrist fractures showed results comparable to the present study. [16, 21]. A recent meta-analysis did, however, not see any significant differences between trained and untrained personnel, supporting the idea that limited training can prove sufficient for this task [12]. All the above-mentioned studies have also used radiographs as a gold standard despite the fact that ED doctors may need to interpret radiographs without the support of a radiologist.

In the present study the aim was to address the above-mentioned limitations. The results also showed that US can be used to distinguish between different types of fractures and subsequently treat them accordingly. The purpose would be to limit the use of radiographs to the cases that may need repositioning or surgery. US was in this study not as good as radiographs for this purpose as seen in the results above. No specific statistical analysis was made for this group as it was obvious from the results presented in the tables.

A more detailed analyses of the misdiagnosed cases shows that the 4/21 complete fractures that should have received a radiographic follow-up (three were considered stable) were all stable without any dislocation at the 1-week radiographic follow-up.

In the present study the sensitivity of the US examination to distinguish fractures needing follow-up from fractures that do not need it is not completely satisfactory if one adheres to the follow-up indications used at the hospital of the study. It is also, however, evident that radiographs are not perfect for this purpose if ED doctors need to interpret the radiographs without the support of a radiologist. Most errors in both groups did, however, result in over-treatment with an unnecessary follow-up rather than under-treatment. The results also serve as a reminder to always properly visualize the distal parts of ulna when examining the wrist.

Since this study evaluates the reliability of US as a triage method to decide which patients needed radiographs, false positives (*n* = 4/116) are of less concern since this group would only need an “unnecessary” radiograph. The same reasoning is valid for the group of uncertain US results (*n*= 7/116).

Point-of-care US in the hands of non-radiologists is a method that has gained scientific support over the last decade and it appears like its use will continue to expand in the ED environment [4, 22–26]. From a global perspective, the World Health Organization has recommended the use of US imaging in developing countries and established minimum specifications for a “general purpose scanner” [27]. In situations like these, the ability to exclude a fracture can be even more valuable and US could also be used to predict the correct treatment, accepting the slightly lower sensitivity compared to radiographs.

### Limitations

The relatively low number of patients included in our study is a limitation as we did not reach the original numbers needed in the pre-study power analysis. Due to the higher than expected accuracy of the US the study group must, however, be considered to be of adequate size to make a valid interpretation. Future studies should further elucidate the field and show the possibilities and limitations of point-of-care ultrasound triage for fractures at the ED.

## Conclusion

Emergency physicians with relevant but limited training can use US to safely rule out a suspected wrist fracture in children and it can significantly decrease the need for radiographs if used as a point-of-care triage tool. US can also be used as a more advanced tool to decide if a suspected fracture warrants a radiograph to decide on further treatment. In the latter case, US examination appears to be less accurate than radiographs as a diagnostic aid for the ED physician.

## Additional file

**Additional file 1.** Ultrasound wrist study.
